# Does COVID-19 infection change appetite and dietary habits?

**DOI:** 10.1097/MD.0000000000050383

**Published:** 2026-09-04

**Authors:** Abeer Salman Alzaben, Anwar Alhashem, Alhnouf M. Albawardi, Alhanouf A. Bin-Saleem, Danya Nawar Alfawaz, Reem I. Alnuwisser, Lujain Almousa

**Affiliations:** a Department of Health Sciences, College of Health and Rehabilitation Sciences, Princess Nourah bint Abdulrahman University, Riyadh, Saudi Arabia.

**Keywords:** appetite, COVID-19, dietary habits, Saudi Arabia, supplement

## Abstract

Coronavirus disease 2019 (COVID-19) has been associated with changes in appetite, taste, and dietary behaviors; however, evidence comparing recovered individuals with those never infected remains limited. Understanding these differences is important for developing post-pandemic nutritional strategies. This study compares appetite characteristics and dietary habits between individuals who recovered from COVID-19 and those who were COVID-free. This cross-sectional study was conducted in January 2022 using an online questionnaire that collected sociodemographic data, medical history, appetite measures, and dietary behaviors. A total of 805 participants were included: 386 recovered from COVID-19 and 419 were COVID-free. Most participants were female (88.3%), with a mean age of 28.60 ± 11.03 years. Group differences were analyzed to identify variations in appetite sensations and eating patterns. Significant differences were observed between groups in fullness (*P* = .044) and taste (*P* < .001), while hunger did not differ significantly. Unhealthy dietary behaviors were common in both groups. Eating more food than planned differed significantly between groups (*P* = .004). Dietary supplement use also varied (*P* = .022), with 8.6% always using supplements, 22.2% sometimes using them, and 53.2% never using them. COVID-19 recovery status was significantly associated with appetite outcomes (*P* < .001). Both groups reported high consumption of junk food and soft drinks and low fruit intake. Recovery from COVID-19 was associated with altered appetite characteristics, particularly fullness and taste. However, unhealthy dietary behaviors were prevalent among both recovered and COVID-free young adults. These findings highlight the need for targeted nutritional interventions and public health strategies to promote healthier eating patterns and support post-pandemic well-being.

**Key points**

- Hunger was similar between the study groups, whereas fullness and taste differed significantly.
- Unhealthy dietary habits, including high consumption of fast food and soft drinks.
- Development of successful nutritional interventions that accelerate recovery and improve overall well-being is required.

## 1. Introduction

Coronavirus disease 2019 (COVID-19) was first detected in December 2019 and has rapidly escalated into a global pandemic. Since early 2020, the world has undergone profound and unprecedented changes in daily life owing to their ongoing impacts, including significant negative health consequences such as fatigue, nausea, and vomiting.^[1]^

Research has identified both short- and long-term complications associated with COVID-19, with a particular emphasis on the persistent loss of taste and smell, which can persist in the post-acute phase.^[2]^ This often results in diminished appetite, which can lead to malnutrition and other long-term health issues.^[2]^ A substantial number of COVID-19 patients have reported a lack of appetite.^[3]^ A cross-sectional study conducted in China found that all participants with COVID-19 reported loss of appetite during their illness.^[4]^

Many individuals have reported a decline in appetite due to symptoms such as fatigue, loss of taste and smell, and gastrointestinal issues associated with COVID-19.^[2–4]^ COVID-19 has been shown to significantly influence eating behavior.^[5]^ Studies have shown that dietary behaviors changed during the COVID-19 pandemic both in Saudi Arabia and globally, including an increase in the consumption of snacks such as biscuits, cakes, soft drinks, and fast foods.^[6,7]^ These dietary shifts not only affect immediate health, but may also have long-term implications for metabolic health and increase the risk of chronic diseases.^[8]^

A recent review, conducted 4 years after the pandemic, reported the long-term effects of COVID-19 infection on several organs, particularly the central nervous system and gastrointestinal system, which are associated with loss of appetite, taste, and smell.^[4]^ The objective of the current study was to assess appetite and dietary habits in individuals who recovered from COVID-19 and those who were COVID-free.

## 2. Methods

### 2.1. Study design and participants

This cross-sectional study was conducted in January 2022. Participants were recruited using snowball sampling. The inclusion criteria were both COVID-19 recovered (COVID group) and COVID-19 free (COVID-free group) adults who lived in Saudi Arabia and were aged 18 to 60 years. Exclusion criteria were chronic diseases that may affect food intake and/or appetite, such as cancer, eating disorders, and kidney diseases.

All participants consented to participate in this study. The study protocol was conducted in accordance with the Declaration of Helsinki and was approved by the Ethics Committee (H-01-R-059), IRB log number 20–0011.

### 2.2. The questionnaire

An online questionnaire link was shared through social media platforms, such as Twitter, Instagram, and WhatsApp. The questionnaire was translated from English into Arabic and then back translated into English to ensure the accuracy of its meaning. This information was reviewed by experts. The final version of the questionnaire was administered to participants in Arabic.

### 2.3. Part 1: Sociodemographic and medical history

Participants were asked to provide sociodemographic information such as age, living region, and living status. Information related to health status, COVID-19 infections, complications, and vaccinations was included in the questionnaire.

### 2.4. Part 2: Appetite and eating habits

Self-reported appetite was assessed using the Simplified Nutritional Appetite Questionnaire,^[9]^ which is validated as a nutritional screening tool and consists of 3 questions related to appetite, hunger, and food taste.

The dietary habits section consisted of 18 questions adapted from previous studies.^[9,10]^ These questions pertained to the number of meals consumed per day on weekdays and weekends, nighttime eating habits, the type and frequency of food consumption, eating behavior, and dietary supplementation. Each question has 4 possible answers.

### 2.5. Statistical analysis

R software version 4.4.0 (R Foundation for Statistical Computing) was used to conduct statistical analyses. The Shapiro–Wilk test and visual examination of Q-Q plots were used to determine normality, and Levene test was used to examine the homogeneity of variances in continuous variables, which were displayed as median interquartile range (IQR). Bivariate analysis was conducted using the Mann–Whitney test because the normality assumptions were unmet. Frequency (%) was used to represent categorical variables. The differences between proportions were assessed using the Freeman–Halton exact test/chi-squared test. Because the study used a modified 3-item version of the Simplified Nutritional Appetite Questionnaire, no validated cutoff score was available. The total 3-item score ranged from 3 to 15. A percentile-based cutoff was used, with participants scoring at or below the 25th percentile of the sample distribution classified as ≤ 10, who were at risk of malnutrition, and those scoring above the 25th percentile, classified as > 10, who were identified as well nourished. We applied binary logistic regression to evaluate the association between COVID-19 status and appetite outcomes and reported the odds ratios (ORs) with 95% confidence intervals (CIs). The total dietary habit score was categorized as low (<35), moderate (35–40), or high (>40). To assess the association between COVID-19 status and dietary habits, we used ordinal logistic regression, the proportional odds assumption of which was tested using the Brant test (*P* = .280). The models were adjusted for age, sex, nationality, province of residence, household composition, and the presence of chronic diseases. Statistical significance was defined as a two-tailed *P*-value of <.05.

## 3. Results

### 3.1. Sociodemographic information

This study included 805 participants, comprising of 386 individuals who recovered from COVID-19 (48.0%) and 419 COVID-free controls (52.0%). Table 1 presents the sociodemographic characteristics of the 2 groups. The participants’ ages ranged from 18 to 60 years in the COVID-recovered group and from 18 to 60 years in the COVID-free group. The COVID-recovered group had a median age of 24 years (IQR = 16.0), whereas the COVID-free group had a median age of 23.0 years (IQR = 10.0).

**Table 1:** Comparison of socio-demographic characteristics between the 2 studied groups.

	Total (n = 805)		COVID recovered (n = 386)		COVID-free (n = 419)		Test of significance	*P*
Age (yrs)**	23 (21–35)		24 (21–37)		23 (21–31)		Mann–Whitney *U* test	**.026** *
	n	%	n	%	n	%		
Gender								
Female	711	88.3	347	89.9	364	86.9	χ^2^= 1.499	.221
Male	94	11.7	39	10.1	55	13.1		
Nationality								
Saudi	763	94.8	363	94.0	400	95.5	χ^2^= 0.561	.454
Non-Saudi	42	5.2	23	6.0	19	4.5		
Province in Saudi Arabia								
Central	686	85.2	342	88.6	344	82.1	χ^2^= 9.289	.054
North	21	2.6	8	2.1	13	3.1		
South	22	2.7	5	1.3	17	4.1		
Eastern	29	3.6	13	3.4	16	3.8		
Western	47	5.8	18	4.7	29	6.9		
Household composition								
Alone	13	1.6	6	1.6	7	1.7	Fisher–Freeman–Halton test	.634
Family	790	98.1	380	98.4	410	97.9		
Friends	2	0.2	0	0.0	2	0.5		

χ2: Chi-square test.

IQR = interquartile range.

** Median and IQR.

* *P*: *P*-value for comparison between the studied groups.

The difference in age between the 2 groups was statistically significant (*P* = .026), with the COVID-recovered group slightly older. The sample was predominantly female in both groups. There were 39 (10.1%) male individuals and 347 (89.9%) female participants in the COVID-recovered group. Similarly, there were 55 males (13.1%) and 364 females (86.9%) in the COVID-free group. The gender distribution did not differ significantly between the groups (*P* = .221). Regarding nationality, 363 (94.0%) participants in the COVID-recovered group and 400 (95.5%) in the COVID-free group were of Saudi nationality. No significant differences were observed between the groups (*P* = .454). Geographically, the participants were predominantly from the Central Region of Saudi Arabia, with 342 COVID-recovered participants (88.6%) and 344 controls (82.1%), totaling to 686 participants (85.2%). Regional distribution did not differ significantly between the groups (*P* = .054). Nearly all participants lived with family members: 380 (98.4%) in the COVID-recovered group and 410 (97.9%) in the COVID-free group. No significant differences were found between the groups (*P* = .634).

### 3.2. Medical history

The prevalence of chronic diseases was examined across the 16 conditions. The 6 most common conditions in the overall sample were obesity (n = 55, 6.8%), diabetes (n = 44, 5.5%), blood pressure disorders (n = 38, 4.7%), asthma (n = 24, 3.0%), eating disorders (n = 20, 2.5%), and thyroid diseases (n = 14, 1.7%). The COVID-recovered group exhibited the following prevalence rates: obesity in 32 participants (8.3%), diabetes in 23 participants (6.0%), blood pressure disorders in 21 participants (5.4%), asthma in 12 participants (3.1%), eating disorders in 7 participants (1.8%), and thyroid diseases in 8 participants (2.1%). The COVID-free group exhibited the following prevalence rates: obesity, 23 participants (5.5%); diabetes, 21 participants (5.0%); blood pressure disorders, 17 participants (4.1%); asthma, 12 participants (2.9%); eating disorders, 13 participants (3.1%); and thyroid diseases, 6 participants (1.4%). Statistical analysis revealed no significant differences between the 2 groups for any of the 16 chronic conditions. The participants who reported food allergies comprised 15% of the COVID-recovered group, whereas the percentage was slightly higher (16.7%) in the COVID-free group; however, the difference was not statistically significant (*P* = .198). Table 2 shows the medical histories of the 2 groups.

**Table 2:** Comparison of medical history between the 2 studied groups.

	Total (n = 805)		COVID recovered (n = 386)		COVID-free (n = 419)		Test of significance	*P*
	No.	%	No.	%	No.	%		
Chronic diseases or conditions you have**								
Obesity	55	6.8	32	8.3	23	5.5	χ^2^ = 2.056	.152
Diabetes	44	5.5	23	6.0	21	5.0	χ^2^ = 0.189	.663
Blood pressure	38	4.7	21	5.4	17	4.1	χ^2^ = 0.575	.448
Asthma	24	3.0	12	3.1	12	2.9	χ^2^ = 0.000	.999
Eating disorders	20	2.5	7	1.8	13	3.1	χ^2^ = 0.897	.343
Thyroid diseases	14	1.7	8	2.1	6	1.4	χ^2^ = 0.180	.671
Food allergy								
Yes	121	15.0	51	13.2	70	16.7	χ^2^ = 1.657	.198
No	684	85.0	335	86.8	349	83.3		
Taking supplement							χ^2^ = 9.651	**.022** *
Always	69	8.6	38	9.8	31	7.4		
Sometimes	179	22.2	101	26.2	78	18.6		
Rarely	129	16.0	55	14.2	74	17.7		
Never	428	53.2	192	49.7	236	56.3		
Following a weight-reduction diet							χ^2^ = 0.053	.818
Yes	95	11.8	44	11.4	51	12.2		
No	710	88.2	342	88.6	368	87.8		

χ^2^: Chi-square test.

** More than 1 answer can be selected.

* *P*: *P*-value for comparing the studied groups.

Regarding supplementation practices, there was a statistically significant difference between the groups (χ2 = 9.651, *P* = .022): the COVID-recovered participants were more likely to report using supplements sometimes (26.2% vs 18.6%), while the COVID-free participants were more likely to report never using supplements (56.3% vs 49.7%). In contrast, the proportion of those following a weight-reduction diet did not differ between the groups (χ2 = 0.053, *P* = .818), with similar proportions reporting yes (11.4% COVID-recovered vs 12.2% COVID-free). Finally, changes in dietary habits after COVID-19 were assessed only among the COVID-recovered individuals, of whom 19.9% reported changes, and 80.1% reported no changes.

### 3.3. COVID-19 infection

Among patients with COVID-19, the distribution of infections has varied over the years. The majority of the patients were infected by 2022 (39.6%), followed by 2021 (31.9%), 2020 (24.4%), and 2019 (4.1%). Regarding reinfection, most participants (90.2%) reported having had only 1 infection, 8.3% were reinfected in 2022, 1.3% in 2021, and a tiny fraction (0.3%) in 2020. Only 4 participants (1.0%) reported having been infected 3 times, with the last infection occurring in 2022. Fever (n = 235, 60.9%), loss of taste (n = 178, 46.1%), loss of smell (n = 196, 50.8%), and diarrhea (n = 72, 18.7%) were the most common COVID-19 complications among the COVID-recovered group. Other notable symptoms included cold/cough/sore throat (n = 59; 15.3%), headache (n = 51; 13.2%), nausea and vomiting (n = 31; 8.0%), bone and muscle pain (n = 30; 7.8%), respiratory pain (n = 28; 7.3%), and fatigue and exhaustion (n = 25; 6.5%). The first, second, and third doses of COVID-19 vaccine were administered to 98.6%, 97.1%, and 40.5% of the participants, respectively.

### 3.4. Appetite

Table 3 compares the appetites of the 2 studied groups. Among all participants, most (40.6%) reported feeling full after eating most of their meals, while 27.7% felt full at the halfway point. A smaller proportion (14.0%) felt full after eating one-third of their meals and 11.2% felt full after just a few bites. There was a statistically significant difference between the 2 groups (*P* = .044), indicating that appetite levels were significantly influenced by the COVID-19 history. Most participants (51.1%) reported feeling hungry at times; 26.0% reported feeling hungry often, and 5.3% reported feeling hungry. No significant difference in hunger sensations was found between the COVID-19-recovered and COVID-free groups (*P* = .090). The majority (54.2%) rated food as excellent, 25.7% as good, and 17.6% as moderate. Only a small proportion of participants described food as bad or very bad. The difference in taste perception between groups was statistically significant (*P* < .001).

**Table 3:** Comparison of appetite between the 2 studied groups.

	Total (n = 805)		COVID recovered (n = 386)		COVID-free (n = 419)		Test of significance	*P*
	No.	%	No.	%	No.	%		
When I eat, I feel full after…								
Few bites	90	11.2	49	12.7	41	9.8	χ^2^ = 9.771	**.044** *
Third of meal	113	14.0	55	14.2	58	13.8		
Half the meal	223	27.7	118	30.6	105	25.1		
Most of meal	327	40.6	136	35.2	191	45.6		
Rarely feel full	52	6.5	28	7.3	24	5.7		
I feel hungry								
Always	43	5.3	23	6.0	20	4.8	χ^2^ = 8.054	.090
Often	209	26.0	95	24.6	114	27.2		
Sometimes	411	51.1	187	48.4	224	53.5		
Rarely	124	15.4	73	18.9	51	12.2		
Don’t feel hungry	18	2.2	8	2.1	10	2.4		
Food tastes…								
Very bad	3	0.4	3	0.8	0	0.0	Fisher–Freeman–Halton test	**<.001** *
Bad	17	2.1	14	3.6	3	0.7		
Middle	142	17.6	91	23.6	51	12.2		
Good	207	25.7	95	24.6	112	26.7		
Excellent	436	54.2	183	47.4	253	60.4		

χ^2^: Chi-square test.

* Statistically significant at *P* ≤ .05. *P*: *P*-value for comparing the studied groups.

### 3.5. Eating habits

Table 4 displays the dietary habits of all participants and the 2 groups studied. Meal consumption patterns showed that 56.3% of participants ate 1 or 2 meals per day, compared to 32.3% who ate 3 or 4 meals per day, 6.1% who ate 5 or 6 meals per day, and 4.3% who ate more than 6 meals per day. These habits remained consistent on weekends, with 49.7% of the participants eating 1 or 2 meals per day and 41.0% eating 3 or 4 meals per day. Additionally, 68.6% of participants never got up at night to eat, compared to 16.8% who rarely ate at night, 12.7% who ate at night, and 2% who always ate at night. Regarding food consumption behaviors, 47.8% reported never continuing to eat after feeling full, 26.8% sometimes ate after feeling full, 37.8% sometimes ate more than planned, and 44.0% sometimes ate when bored. Emotional eating was reported by 13.8% of participants who always ate when feeling depressed or unhappy, with 15.4% consistently consuming red meat, 62.5% consistently consuming chicken, and 10.4% consistently consuming fish. When examining nutritional choices, 53.8% of the participants consumed full-fat milk, while 60.4% preferred white bread to whole wheat bread (36.5%). Fruit intake was generally low, with 40.7% not consuming fruit at all and only 0.6% consuming more than 5 servings per day. Additionally, 33.8% rarely consumed soft or energy drinks, whereas 59.6% sometimes purchased fast food.

**Table 4:** Comparison of dietary habits between the 2 studied groups.

	Total (n = 805)		COVID recovered (n = 386)		COVID-free (n = 419)		Test of significance	*P*
	No.	%	No.	%	No.	%		
Number of meals per day on weekdays								
1–2 meals	461	57.3	222	57.5	239	57.0		
3–4 meals	260	32.3	129	33.4	131	31.3	χ^2^ = 1.692	.639
5–6 meals	49	6.1	21	5.4	28	6.7		
More than 6 meals	35	4.3	14	3.6	21	5.0		
Number of meals per day on the weekend								
1–2 meals	400	49.7	197	51.0	203	48.4		
3–4 meals	330	41.0	160	41.5	170	40.6	χ^2^ = 3.569	.312
5–6 meals	64	8.0	26	6.7	38	9.1		
More than 6 meals	11	1.4	3	0.8	8	1.9		
Get up at night to eat								
Always	16	2.0	5	1.3	11	2.6		
Sometimes	102	12.7	45	11.7	57	13.6	χ^2^ = 2.979	.395
Rarely	135	16.8	63	16.3	72	17.2		
Never	552	68.6	273	70.7	279	66.6		
Continuing to eat after feeling full								
Always	25	3.1	11	2.8	14	3.3		
Sometimes	216	26.8	93	24.1	123	29.4	χ^2^ = 7.516	.057
Rarely	179	22.2	78	20.2	101	24.1		
Never	385	47.8	204	52.8	181	43.2		
Eating more food than planned								
Always	57	7.1	28	7.3	29	6.9		
Sometimes	304	37.8	130	33.7	174	41.5	χ^2^ = 13.359	.004*
Rarely	212	26.3	94	24.4	118	28.2		
Never	232	28.8	134	34.7	98	23.4		
Eating food when you feel bored								
Always	141	17.5	63	16.3	78	18.6		
Sometimes	354	44.0	172	44.6	182	43.4	χ^2^ = 1.202	.752
Rarely	156	19.4	79	20.5	77	18.4		
Never	154	19.1	72	18.7	82	19.6		
Eating when depressed or unhappy								
Always	111	13.8	47	12.2	64	15.3	χ^2^ = 2.671	.445
Sometimes	235	29.2	116	30.1	119	28.4		
Rarely	165	20.5	75	19.4	90	22.5		
Never	294	36.5	148	38.3	146	34.8		
Eating red meat								
Always	124	15.4	57	14.8	67	16.0	χ^2^ = 0.501	.919
Sometimes	402	49.9	196	50.8	206	49.2		
Rarely	143	17.8	70	18.1	73	17.4		
Never	136	16.9	63	16.3	73	17.4		
Eating chicken								
Always	503	62.5	242	62.7	261	62.3	χ^2^ = 1.192	.755
Sometimes	255	31.7	119	30.8	136	32.5		
Rarely	34	4.2	17	4.4	17	4.1		
Never	13	1.6	8	2.1	5	1.2		
Eating fish								
Always	84	10.4	42	10.9	42	10.0	χ^2^ = 1.458	.692
Sometimes	312	38.8	154	39.9	158	37.7		
Rarely	305	37.9	138	35.8	167	39.9		
Never	104	12.9	52	13.5	52	12.4		
Milk consumption								
Full fat	433	53.8	211	54.7	222	53.0	χ^2^ = 0.631	.889
Low fat	195	24.2	92	23.8	103	24.6		
Fat free	13	1.6	5	1.3	8	1.9		
Do not consume milk	164	20.4	78	20.2	86	20.5		
Bread consumption								
White	486	60.4	232	60.1	254	60.6	χ^2^ = 0.173	.917
Whole wheat bread	294	36.5	141	36.5	153	36.5		
Do not consume bread	25	3.1	13	3.4	12	2.9		
Fruit consumption								
1–2 servings	449	55.8	215	55.7	234	55.8	Fisher–Freeman–Halton test	.598
3–4 servings	23	2.9	8	2.1	15	3.6		
More than 5 servings	5	.6	3	0.8	2	0.5		
Do not consume fruit	328	40.7	160	41.5	168	40.1		
Consuming a soft or energy drink								
Always	94	11.7	44	11.4	50	11.9	χ^2^ = 2.528	.470
Sometimes	243	30.2	116	30.1	127	30.3		
Rarely	272	33.8	123	31.9	149	35.6		
Never	196	24.3	103	26.7	93	22.2		
Purchasing fast food or takeaways								
Always	115	14.3	52	13.5	63	15.0	χ^2^ = 1.443	.696
Sometimes	480	59.6	236	61.1	244	58.2		
Rarely	173	21.5	83	21.5	90	21.5		
Never	37	4.6	15	3.9	22	5.3		

χ^2^: Chi-square test.

* Statistically significant at *P* ≤ .05. *P*: *P*-value for comparing between the studied groups.

Dietary behaviors were generally similar between COVID-19 recovered and COVID-free participants. There was no significant difference in the number of meals consumed daily on weekdays (*P* = .639) and weekends (*P* = .312). Additionally, there were no significant differences in eating habits at night (*P* = .395), continuing to eat after feeling full (*P* = .057), eating while bored (*P* = .752), or eating when depressed or unhappy (*P* = .445). COVID-free participants reported eating more food than planned (*P* = .004), whereas the consumption of red meat (*P* = .919), chicken (*P* = .755), fish (*P* = .692), milk (*P* = .889), bread (*P* = .917), fruit (*P* = .598), soft/energy drinks (*P* = .470), and fast food (*P* = .696) did not differ between the groups.

Notably, there was a significant difference in the use of dietary supplements between the groups (*P* = .022), with 8.6% of participants always taking supplements, 22.2% sometimes taking supplements, and 53.2% never taking supplements. However, only 11.8% of the participants were actively on a weight reduction diet, with no significant association with COVID-19 history. Lastly, 19.9% of individuals who had COVID-19 reported changing their dietary habits after infection, compared with no changes among the participants who were COVID-free.

### 3.6. Characteristics associated with appetite

Table 5 displays the outcomes of the logistic regression model for variables associated with appetite. The COVID-19 recovery status was significantly associated with appetite outcomes. In both the univariate (OR = 0.54, 95% CI: 0.41–0.72, *P* < .001) and multivariate (OR = 0.56, 95% CI: 0.42–0.74, *P* < .001) models, participants who had recovered from COVID-19 were less likely to report being well-nourished than those who were COVID-free. The appetite outcome was inversely related to age, with a 2% decrease in the odds of being well nourished for every year of age (univariate OR = 0.98, 95% CI: 0.97–0.99, *P* = .002; multivariate OR = 0.98, 95% CI: 0.97–0.99, *P* = .022). In the univariate analysis, chronic disease was also associated with a decrease in the odds of being well nourished (OR = 0.65, 95% CI: 0.46–0.92, *P* = .014); however, this association was eliminated after adjustment (OR = 0.73, 95% CI: 0.51–1.05, *P* = .089). Other factors that were not significantly correlated with appetite were sex, nationality, province of residence, and household composition.

**Table 5:** Predictors of appetite (logistic regression analysis).

	Univariate			Multivariate		
	OR	95% CI	*P*-value	OR	95% CI	*P*-value
Group						
COVID free	–	–		–	–	
COVID recovered	0.54	0.41–0.72	**<.001** *	0.56	0.42–0.74	**<.001** *
Age	0.98	0.97–0.99	**.002** *	0.98	0.97–0.99	**.022** *
Gender						
Female	–	–		–	–	
Male	1.09	0.70–1.69	.705	1.15	0.73–1.82	.551
Nationality						
Non-Saudi	–	–		–	–	
Saudi	1.54	0.83–2.87	.174	1.47	0.78–2.79	.232
Province in Saudi Arabia						
Center	–	–		–	–	
Other provinces	1.10	0.74–1.63	.649	0.99	0.66–1.49	.966
Household composition						
No	–	–		–	–	
Family	0.91	0.32–2.58	.860	1.10	0.37–3.24	.862
Chronic diseases	0.65	0.46–0.92	**.014** *	0.73	0.51–1.05	.089

CI = confidence interval, OR = odds ratio.

* *P* < .05.

### 3.7. Characteristics associated with dietary habits

The results of the ordinal logistic regression model are presented in Table 6. Dietary habit categories were not significantly associated with COVID-19 recovery status in univariate analysis (OR = 1.17, 95% CI: 0.90–1.52, *P* = .244). This association remained nonsignificant (OR = 1.05, 95% CI: 0.80–1.38, *P* = .704) after adjusting for covariates. Age was significantly associated with higher odds of being in a higher dietary habit category, with each 1-year increase in age associated with a 6% increase in odds in the univariate model (OR = 1.06, 95% CI: 1.05–1.08, *P* < .001) and a 7% increase after adjustment (OR = 1.07, 95% CI: 1.05–1.08, *P* < .001). Other variables, including sex, nationality, province of residence, household composition, and presence of chronic disease, were not significantly associated with the dietary habit categories.

**Table 6:** Predictors of dietary habits (ordinal logistic regression analysis).

	Univariate			Multivariate		
	OR	95% CI	*P*-value	OR	95% CI	*P*-value
Group						
COVID free	–	–		–	–	
COVID recovered	1.17	0.90–1.52	.244	1.05	0.80–1.38	.704
Age	1.06	1.05–1.08	<.001*	1.07	1.05–1.08	<.001*
Gender						
Female	–	–		–	–	
Male	1.10	0.73–1.64	.654	0.87	0.57–1.33	.520
Nationality						
Non-Saudi	–	–		–	–	
Saudi	0.63	0.34–1.17	.145	0.71	0.38–1.31	.271
Province in Saudi Arabia						
Center	–	–		–	–	
Other provinces	1.04	0.72–1.50	.815	1.17	0.80–1.71	.420
Household composition						
No	–	–		–	–	
Family	1.30	0.51–3.36	.578	1.02	0.38–2.70	.968
Chronic diseases	1.20	0.87–1.67	.265	0.74	0.52–1.04	.088

CI = confidence interval, OR = odds ratio.

* *P* < .05.

## 4. Discussion

This study was conducted after the COVID-19 lockdown to compare the dietary habits and appetite of young adults who recovered from COVID-19 and young COVID-free individuals living in Saudi Arabia. In general, self-reported COVID-19 recovery status was significantly associated with appetite outcome. Both groups reported unhealthy dietary habits (high consumption of junk food and soft drinks and low consumption of fruit). Individuals who recovered from COVID-19 took more supplements than those in the COVID-free group did.

Several biological mechanisms may explain the observed changes in appetite and dietary habits following COVID-19.^[11–13]^ Persistent olfactory and gustatory dysfunctions are among the most frequently reported sequelae of COVID-19 and may alter food preferences, reduce food enjoyment, and affect overall dietary intake. Furthermore, SARS-CoV-2-related inflammation may disrupt the neural pathways involved in hunger and satiety regulation, potentially influencing appetite and eating behaviors. Emerging evidence also suggests that post-COVID neurological alterations and disturbances in gut–brain signaling may contribute to long-term changes in feeding behavior. A better understanding of these mechanisms may support the development of targeted interventions including nutritional counseling, sensory rehabilitation, and individualized dietary strategies for individuals experiencing persistent post-COVID symptoms. The current study did not assess the mechanisms of changes in appetite during COVID. It is important to emphasize the need for longitudinal studies that incorporate inflammatory biomarkers and objective nutritional assessments. This result is consistent with that of the current study, which found no differences between the study groups in terms of their feelings of hunger. Moreover, Chaaban et al^[14]^ found that the COVID-19 infection led to long-term decreased feelings of hunger and immediate feelings of fullness. This study also noted changes in chemosensory perception that contribute to reduced appetite. Likewise, the current study found a significant difference between the studied groups in terms of feeling full and tasting, with approximately 28% of COVID-19 patients reporting that food tasted middle or bad. Similarly, a previous study reported that COVID-19 patients may experience a reduced sense of smell and taste, which may affect their appetite.^[14]^ In Saudi Arabia, almost 42% of participants who recovered from COVID-19 experienced loss of appetite.^[15]^ An Iranian investigation also demonstrated that lack of appetite was a common symptom reported by patients who had recovered from COVID-19.^[16]^ This shows that the organ systems implicated in the result related to the association between feeling hunger and fullness and the sense of smell and taste in individuals with COVID-19 have not been clearly identified.^[4]^

Several studies have reported numerous dietary habits among individuals with COVID-19 and/or during the COVID-19 lockdown.^[5–7]^ Our findings showed no significant differences between the 2 groups in terms of the number of daily meals consumed. Notably, on the weekends, both groups had an increased number of meals. In contrast, Ismail et al^[17]^ claimed that there was an increase in the daily meals consumed during the COVID-19 pandemic, especially breakfast, which led to weight gain. Similarly, a UK survey found that more than half of their sample increased the number of snacks consumed during the COVID-19 pandemic.^[18]^ A study conducted in the Kurdish region found that almost 33% of the participants consumed more meals and snacks after the COVID-19 pandemic^[19]^

Nearly two-thirds of the respondents in our sample indicated that they rarely or never continued eating after feeling full. In addition, more than 68% indicated that they never got up at night to eat, with coronavirus infection having no effect on this behavior. It is interesting to note that there was a significant difference between the 2 study groups in terms of eating more food than planned, whereas over one-third of the COVID-recovered group sometimes consumed more food. A cross-sectional study involving 1964 voluntary participants reported that more than 30% of participants consumed more food, primarily carbohydrate-rich food, after contracting COVID-19.^[20]^ This increase in food consumption may be attributed to the fact that humans under stress and pressure tend to consume more food, particularly empty-calorie food, to feel calm.^[21]^

A study conducted in Italy on the effects of COVID-19 on food consumption found that approximately 50% of the study sample ate more food, with only 21% opting for healthier choices.^[22]^ This is in agreement with the study by Di Renzo et al,^[23]^ who reported that throughout the COVID-19 lockdown in Italy, more than one-third of the population experienced increased appetite, while approximately 18% suffered from a lack of appetite, indicating that feelings of satiety and hunger had shifted in almost half of the population.^[23]^ A similar finding was observed in Australia, where a study found that approximately 50% of the study population mentioned either having increased appetite or a lack of appetite,^[24]^ whereas a survey of the Kurdish population estimated that more than 30% of participants had gained weight due to increased appetite.^[19]^ Inceu et al^[5]^ reported that increased appetite results from higher ghrelin levels in COVID-19-infected individuals.

An analysis of the effect of COVID-19 on emotional eating found that approximately 44% of the participants in both groups sometimes ate when they were bored, although more than 38% of the participants in both groups rarely or never ate when they were bored. Our results are also consistent in that approximately one-third of the participants in both groups sometimes ate when they were depressed or unhappy, whereas more than 57% rarely or never ate when they were depressed or unhappy. This study found no significant association between COVID-19 infection and emotional eating, a result that is consistent with a previous study conducted in Spain that found that COVID-19 did not influence obese Spanish adults and did not result in Spanish adults developing eating disorders.^[25]^ However, many studies have suggested that depression, anxiety, and stress increase emotional eating.^[20]^ Rodriguez-Moreno et al^[26]^ found that the stress resulting from COVID-19 altered eating behavior, either by increasing appetite or by leading to a lack of appetite.

The results of our survey showed that a considerable proportion of participants reported changes in their dietary habits after COVID-19, suggesting that the effects of COVID-19 on eating behaviors may persist beyond the acute phase of the illness. These changes may be related to alterations in taste and smell, persistent symptoms, or other physiological and behavioral factors associated with post-COVID recovery. Further longitudinal studies are needed to examine the long-term effects of COVID-19 on appetite regulation, food preferences, dietary behaviors, and supplement use as well as to identify the underlying mechanisms. A better understanding of these relationships may help to inform evidence-based nutritional interventions and public health strategies to support individuals recovering from COVID-19. In agreement with our observations, Poelman et al^[27]^ found that most Dutch adult participants in their study did not change their eating habits during the pandemic. In contrast, many studies have found that most of their samples changed their dietary habits, leading to unhealthy dietary patterns and weight gain during the lockdown.^[17–19,28–30]^ In addition, changes in dietary habits, dominated by unhealthy dietary patterns, remained in 88.6% of the samples after quarantine in Saudi Arabia.^[31]^

The results of the current study did not show a marked difference between the study groups in terms of consumption of different types of food after COVID-19. In general, the results indicated that 50%, 39%, 60%, and 30% of the participants sometimes consumed meat, fish, fast food, and soft or energy drinks, respectively, whereas 62% always consumed chicken, 54% consumed full-fat milk, and 20% did not consume milk. A total of 60% ate white bread, 56% ate fruit once or twice daily, and 41% did not consume any fruits. The remaining participants’ percentages were distributed among rare, never, and always responses, indicating diverse dietary patterns across the food items. Supporting these findings, a 2020 study in the UAE by Ismail et al noted that 50% of their sample did not eat fruits daily and 30% did not drink milk.^[17]^ Di Renzo et al^[23]^ conducted their study in Italy and have reported similar results. The participants’ consumption of fruits, vegetables, and fish decreased, while their consumption of junk food increased during the COVID-19.^[23]^ A study in Tunisia found that only 21% of the sample consumed more fruit than usual during COVID-19, while 36% drank more sugary drinks.^[30]^ A study in Saudi Arabia found that the pandemic led to unhealthy dietary habits, with 33% of participants eating fast food and 30% eating high-sugar foods, with only 34% increasing their intake of fruits and vegetables and decreasing their intake of fast food.^[31]^ In contrast, a 2022 study by Marty et al reported that during COVID-19 in France, participants increased their consumption of fruits and fish.^[32]^

Although the prevalence of obesity, diabetes mellitus, high blood pressure, and thyroid disease was higher among participants in the COVID-recovered group than among those in the COVID-free group, these differences were not statistically significant. The observed pattern may reflect differences in lifestyle, socioeconomic factors, and access to health care. Alternatively, a previous COVID-19 infection may contribute to the development or progression of chronic conditions through persistent inflammation, oxidative stress, or alterations in the gut microbiome. Accumulating evidence suggests that SARS-CoV-2 infection may contribute to persistent metabolic alterations through chronic inflammation, immune dysregulation, oxidative stress, and gut microbiome disturbances, which have been implicated in obesity, insulin resistance, and diabetes.^[33,34]^ Further longitudinal studies are needed to clarify the temporal relationships among COVID-19 infection, chronic diseases, and dietary habits and to better understand the underlying mechanisms that may inform targeted interventions.

It is worth mentioning that the results showed that more than half of the study participants never took supplements, whereas only 9% always took supplements. A study conducted in Turkey found that 35% of study participants started taking supplements during COVID-19.^[35]^ This is similar to a study conducted in Iran, which reported that participants increased their intake of supplements to enhance their immunity to COVID-19.^[16]^ This difference may reflect increased health awareness or changes in health-seeking behaviors following infection, as individuals recovering from COVID-19 may be more inclined to adopt practices that they perceive as beneficial to their health. These findings suggest that the relationship between COVID-19 and dietary behaviors is complex and warrants further investigation.

Most studies have assessed dietary habits during the COVID-19 lockdown or in young individuals who were infected with COVID-19, with only a limited number of studies comparing appetite and dietary habits between young individuals who recovered from COVID-19 and those who had COVID-19. Although this was a strength of the present study, its limitations should be noted. This study relied on self-reported data, which may have been subject to a reporting bias. Participants were asked to recall their dietary habits before COVID-19, introducing the potential for recall bias. The majority of the study sample was female, which is common because of the study design and sampling technique.^[6]^ Although the COVID-recovered and COVID-free groups were comparable with respect to measured sociodemographic and medical history variables, residual confounding from unmeasured socioeconomic and lifestyle factors may have influenced the observed associations. Another limitation is related to the timing of data collection. Most respondents had recovered from COVID-19 more than 1 year before participation, which can decrease the chances of detecting differences in appetite or taste between the groups. Therefore, it was not possible to analyze the potential impact of specific supplement types on appetite or dietary habits.^[36]^ Thus, the manuscript does not provide sufficient information regarding the types of supplements used and their possible impacts on appetite and dietary habits. The study population consisted predominantly of young adults; therefore, the findings may not be generalizable to older populations who may be more vulnerable to COVID-19 and may exhibit different appetite- and diet-related responses following COVID-19. Future studies should include older participants and more detailed questions on supplement use to better understand its influence on nutritional behavior. Another limitation was that changes in dietary habits after COVID-19 were assessed using a binary yes/no question. Therefore, this study could not determine the specific types of dietary changes reported, such as changes in meal frequency, portion size, food group intake, or dietary quality. In addition, because of the cross-sectional design, the relationship between these reported changes, appetite outcomes, and overall health outcomes could not be determined causally.

## 5. Conclusion

Self-reported appetite was significantly different between the study groups in terms of feeling full and food taste. Unhealthy dietary habits, such as high consumption of fast foods and soft drinks and low consumption of fruit, were prevalent in both the COVID and COVID-free groups. Developing successful nutritional interventions aimed at accelerating recovery and improving general well-being requires understanding the connection between COVID-19 and dietary practices. Further research is necessary to address these public health and nutrition issues.

## Author contributions

**Conceptualization:** Abeer Salman Alzaben, Anwar Alhashem, Alhnouf M. Albawardi, Alhanouf A. Bin-Saleem, Danya Nawar Alfawaz, Reem I. Alnuwisser, Lujain Almousa.

**Data curation:** Abeer Salman Alzaben, Anwar Alhashem.

**Formal analysis:** Abeer Salman Alzaben, Anwar Alhashem, Lujain Almousa.

**Funding acquisition:** Abeer Salman Alzaben.

**Investigation:** Abeer Salman Alzaben.

**Methodology:** Abeer Salman Alzaben, Anwar Alhashem, Alhnouf M. Albawardi, Alhanouf A. Bin-Saleem, Danya Nawar Alfawaz, Reem I. Alnuwisser, Lujain Almousa.

**Project administration:** Abeer Salman Alzaben.

**Resources:** Abeer Salman Alzaben, Anwar Alhashem, Lujain Almousa, Anwar Alhashem, Alhnouf M. Albawardi, Alhanouf A. Bin-Saleem, Danya Nawar Alfawaz, Reem I. Alnuwisser.

**Supervision:** Abeer Salman Alzaben.

**Validation:** Abeer Salman Alzaben, Anwar Alhashem, Lujain Almousa.

**Writing – original draft:** Abeer Salman Alzaben, Anwar Alhashem, Lujain Almousa.

**Writing – review & editing:** Abeer Salman Alzaben, Anwar Alhashem, Lujain Almousa.
